# Monitoring levels of circulating cell‐free DNA in patients with metastatic colorectal cancer as a potential biomarker of responses to regorafenib treatment

**DOI:** 10.1002/1878-0261.12972

**Published:** 2021-06-22

**Authors:** Brice Pastor, Thierry André, Julie Henriques, Isabelle Trouilloud, Christophe Tournigand, Marine Jary, Thibault Mazard, Christophe Louvet, Simon Azan, Audrey Bauer, Benoit Roch, Cynthia Sanchez, Dewi Vernerey, Alain R. Thierry, Antoine Adenis

**Affiliations:** ^1^ Institut de Recherche en Cancérologie de Montpellier (IRCM) INSERM Université de Montpellier Montpellier Cancer Institute (ICM) France; ^2^ Department of Medical Oncology Saint‐Antoine University Hospital Sorbonne University Paris France; ^3^ Oncology Multidisciplinary Research Group (GERCOR) Paris France; ^4^ Methodology and Quality of Life Unit in Oncology Besançon University Hospital France; ^5^ Medical Oncology Service Henri Mondor Hospital AP‐HP Université Paris Est Créteil Créteil France; ^6^ INSERM Etablissement Français du Sang Bourgogne Franche‐Comté UMR1098, Interactions Hôte‐Greffon‐Tumeur/Ingénierie Cellulaire et Génique Bourgogne Franche‐Comté University Besançon France; ^7^ Department of Medical Oncology Besançon University Hospital France; ^8^ Department of Medical Oncology Montpellier Cancer Institute (ICM) France; ^9^ Department of Medical Oncology Institut Monsouris Paris France; ^10^ Department of Thoracic Oncology Montpellier University Hospital Université de Montpellier France

**Keywords:** circulating DNA, colorectal cancer, predictive biomarker, regorafenib, tumor response

## Abstract

Circulating cell‐free DNA (cfDNA) contains circulating tumor DNA (ctDNA), which can be obtained from serial liquid biopsies to enable tumor genome analysis throughout the course of treatment. We investigated cfDNA and mutant ctDNA as potential biomarkers to predict the best outcomes of regorafenib‐treated metastatic colorectal cancer (mCRC) patients. We analyzed longitudinally collected plasma cfDNA of 43 mCRC patients prospectively enrolled in the phase II TEXCAN trial by IntPlex qPCR. Qualitative (*KRAS*, *NRAS, BRAF^V600E^* mutations) and quantitative (total cfDNA concentration, mutant ctDNA concentration, mutant ctDNA fraction) parameters were correlated with overall survival (OS) and progression‐free survival (PFS). When examined as classes or continuous variables, the concentrations of total cfDNA, mutant ctDNA, and, partly, mutant ctDNA fraction prior to regorafenib treatment correlated with OS. Patients with baseline cfDNA > 26 ng·mL^−1^ had shorter OS than those with cfDNA value below this threshold (4.0 vs 6.9 months; log‐rank *P* = 0.0366). Patients with baseline mutant ctDNA > 2 ng·mL^−1^ had shorter OS than those with mutant ctDNA below this threshold (log‐rank *P* = 0.0154). We show that pretreatment cfDNA and mutant ctDNA levels may identify mCRC patients that may benefit from regorafenib treatment.

## Graphical abstract

Circulating cell‐free DNA (cfdNA) was extracted from longitudinal blood collection of 43 mCRC patients treated by regorafenib. Qualitative (*RAS, BRAF^V600E^* mutations) and quantitative (cfDNA concentration, mutant circulating tumor DNA (ctDNA) concentration, mutant ctDNA fraction) parameters were correlated with survival. We show that pretreatment cfDNA and mutant ctDNA levels may identify mCRC patients that may benefit from regorafenib treatment.

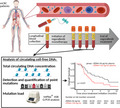

**Abbreviations**
95% CI: 95% confidence interval
C1: cycle 1
C2: cycle 2
cfDNA: circulating cell‐free DNA
CRC: colorectal cancer
ctDNA: circulating tumor DNA
ECOG PS: Eastern Cooperative Oncology Group performance status
EGFR: epidermal growth factor receptor
EOT: end of treatment
HR: hazard ratio
IQR: interquartile range
mCRC: metastatic colorectal cancer
OS: overall survival
PFS: progression-free survival
RECIST: response evaluation criteria in solid tumors
WT: wild‐type

## 1 Introduction

Colorectal cancer (CRC) is a major cause of morbidity and mortality globally [1]. In 2018, there were nearly 2 million newly diagnosed patients and almost 880 792 deaths. Approximately 50% of patients develop metastasis, and most of them require palliative treatment. In that setting, patients with metastatic CRC (mCRC) are treated, concomitantly or sequentially, with different cytotoxic agents such as fluoropyrimidines, oxaliplatin, and irinotecan that may be combined with monoclonal antibodies against vascular endothelial growth factor or epidermal growth factor receptor (EGFR) [2]. The latter combination is restricted to patients with *KRAS* and *NRAS* (*RAS*) wild‐type (WT) tumors. In otherwise standard therapies‐refractory mCRC patients, oral agents such as trifluridine/tipiracil or regorafenib could be considered [2].

Regorafenib is a multityrosine kinase inhibitor that induces *in vitro* and *in vivo* anti‐angiogenic and cytotoxic activities [3]. It was approved in the European Union for refractory mCRC based on the report of its activity versus placebo plus best supportive care in the CORRECT and CONCUR phase III clinical trials [4, 5]. In these two studies, regorafenib demonstrated a significant improvement in overall survival (OS) in patients with mCRC who progressed on standard therapies [4, 5]. However, half of the treated patients experienced tumor progression 2 months after starting treatment, which unnecessarily exposed them to treatment‐related adverse events. That toxicity risk could be reduced by adopting a dose‐escalation strategy for optimizing regorafenib dosing with comparable activity [6]. In an exploratory study of the pivotal CORRECT trial [4], Tabernero *et al*. (2015) showed a trend toward a better survival for patients treated with regorafenib who presented *KRAS* or *PIK3CA* WT genotypes, assessed using tissue DNA or circulating cell‐free DNA (cfDNA). Interestingly, they also observed an association between high baseline cfDNA and reduced median OS [7]. Subsequently, in a retrospective study of 654 patients with mCRC who received regorafenib in a compassionate use program reported by Adenis *et al*. [8] OS was independently and unfavorably affected by poor performance status, short time from initial diagnosis of metastasis to the start of regorafenib treatment, low initial regorafenib dose, > 3 metastatic sites, the presence of the liver metastasis, and tissue *KRAS* mutations. However, there are currently no widely accepted criteria predicting benefits from regorafenib.

Circulating cell‐free DNA is a plasma biomarker widely used in oncology [9], notably for detecting *RAS/BRAF* point mutations before the initiation of anti‐EGFR agents in mCRC patients [10, 11, 12, 13, 14, 15]. Moreover, longitudinally analysis of cfDNA was used to address the temporal and spatial clonal heterogeneity of mCRC patients [16, 17, 18, 19, 20, 21].

Phase II TEXCAN trial aimed to evaluate tumor response in refractory, progressing mCRC patients after 2 months of treatment with regorafenib [22]. In the present study, we investigated the clinical activity of regorafenib in those patients according to analysis of circulating cfDNA.

## 2 Materials and method

### 2.1 Patients and study design

TEXCAN (NCT02699073) was a multicenter, prospective, open‐label, single‐arm phase II trial of patients with mCRC refractory to standard therapy. The study aimed to evaluate the 2‐month tumor response to treatment with regorafenib (160 mg once daily for 3 weeks, followed by 1 week off therapy) using different measurement methods. Inclusion/exclusion criteria, study design, study objectives/endpoints, and clinical results have been previously reported [22]. Briefly, 55 patients (the intent‐to‐treat population) received at least one regorafenib tablet (1 tablet = 40 mg). According to response evaluation criteria in solid tumors (RECIST) 1.1, no partial and complete responses were observed. The best overall response observed was stable disease in 20/35 patients evaluable for the primary endpoint (i.e., a 2‐month clinical response). The median OS was 5.3 months (95% confidence interval [CI]: 3.7–8.6). Unlike RECIST, other measurement methods, specifically Choi and modified Choi criteria, did not identify any survival benefit for mCRC patients [22].

**Fig. 1:**
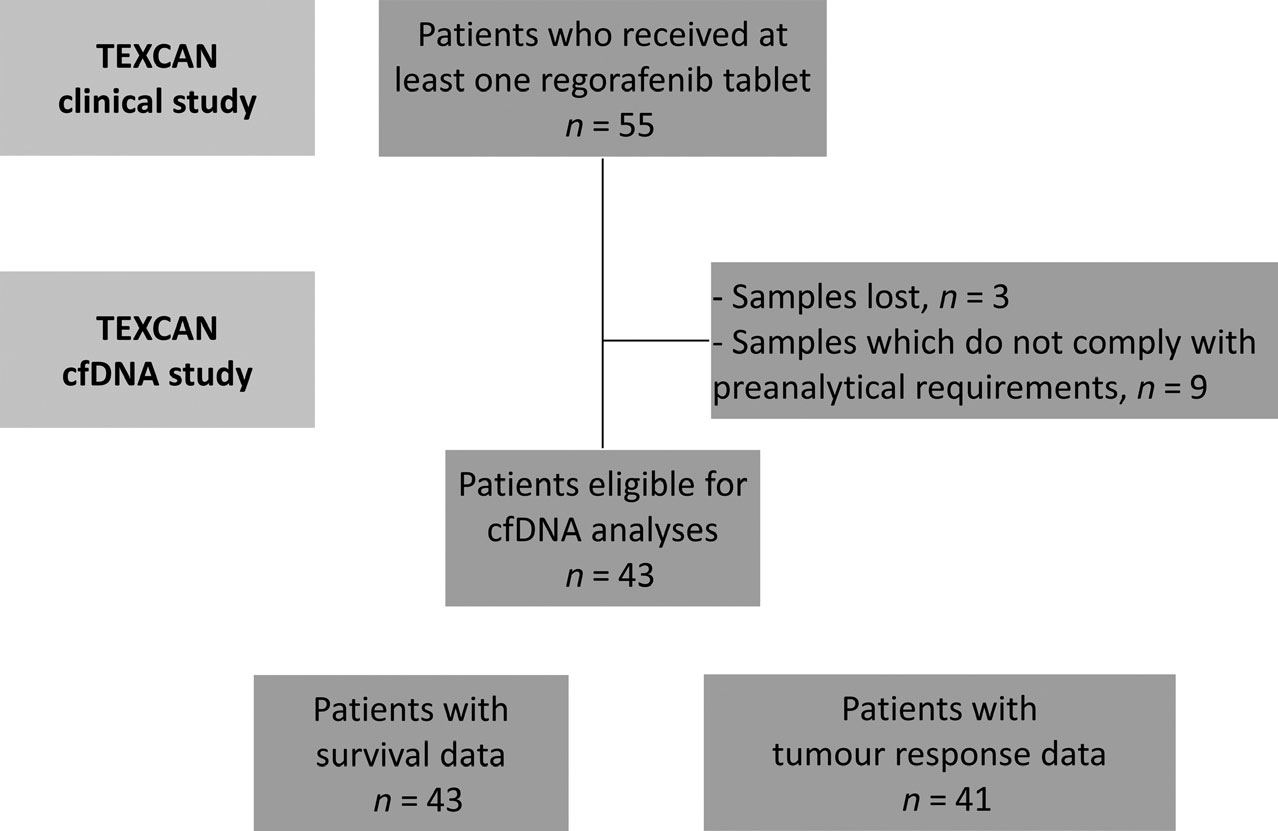
Flowchart. 55 patients (the intent‐to‐treat population) received at least one regorafenib tablet. 43 out of 55 patients had survival data, and 41 out of 55 had tumor response data. Patients with survival and tumor response data had also circulating cell‐free DNA data.

The trial was submitted and approved by French regulatory authorities (Agence Nationale de Sécurité du Médicament et des Produits de Santé and the Comité de Protection des Personnes Ile de France VI [French Ethics Committee]; it complies with the Declaration of Helsinki and local regulations, and follows the standards of Good Clinical Practice (ICH‐E6 R2). All patients provided a written informed consent. An exploratory analysis aimed (a) to determine whether a correlation exists between baseline cfDNA levels and survival outcomes, (b) to evaluate the dynamic of total cfDNA during treatment, and (c) to determine the *RAS/BRAF* mutational status in plasma samples, at baseline and at the end of treatment (EOT). Of 55 included patients, 12 were excluded because their baseline samples failed to comply with our pre‐analytical requirements (*n* = 9) [23, 24] or lost during transportation (*n* = 3), leaving 43 eligible patients for cfDNA analysis (the cfDNA population; Fig. 1).

### 2.2 Sample preparation

Blood samples were collected between March 2016 and July 2017 in ethylenediaminetetraacetic acid tubes and centrifuged at 1200 ***g*** for 10 min at 4 °C, within 4 h after collection. Blood draws for cfDNA analysis were scheduled at baseline (before regorafenib therapy), during treatment (day 15 of cycles 1 and 2), and at the EOT. Plasma samples were immediately stored at 80 °C and transferred on dry ice from the recruiting institutions to our laboratory. Plasma was stored few months (4–24 months), and plasma was centrifuged at 16 000 ***g*** for 10 min at 4 °C. Total cfDNA was extracted from 1 mL of plasma using the QIAamp DNA Mini Blood Kit (Qiagen, Hilden, Germany) in accordance with the previously published pre‐analytic guidelines [23, 24] in an elution volume of 130 µL. DNA extracts were kept few days (1 to 3 days) at −20 °C until use if not used immediately. Circulating cfDNA was also extracted from 1 mL plasma (Maxwell® RSC Instrument) using the cfDNA Plasma Kit (Promega Corporation, Madison, WI, USA) in an elution volume of 130 µL. In total, 136 serial plasma samples from 43 patients were analyzed. We strictly followed the pre‐analytical requirements to quantify cfDNA under the best conditions (plasma isolation, plasma storage at −80 °C; [23, 24]). No significant variation of cfDNA concentration as determined by the WT sequence of the *KRAS* (67 bp length) was found upon storage at −80 °C [23, 24, 25] for up to 3 years. We performed analysis of all pre‐analytical factors such as storage.

### 2.3 IntPlex® analysis of cfDNA

Analysis of cfDNA was done by IntPlex®, an allele‐specific blocker quantitative PCR (ASB qPCR), which we previously described [10, 21, 26], according to the MIQE guidelines [27, 28]. This IntPlex system specifically detect nuclear cfDNA. qPCR amplifications were carried out at least in two replicates in a total volume of 25 µL on a CFX96 instrument using the CFX manager software (Bio‐Rad, Hercules, CA, USA). Each PCR was composed of 12.5 µL of IQ Supermix SYBR Green (Bio‐Rad), 2.5 µL of DNase‐free water (Qiagen) or specific oligoblocker, 2.5 µL of forward and reverse primers (0.3 pmol·mL^−1^), and 5 µL of template. Thermal cycling comprised three repeated steps: a hot start activation step at 95 °C for 3 min, followed by 40 cycles of denaturation–amplification at 95 °C for 10 s, and then at 60 °C for 30 s. Melting curves were investigated by increasing the temperature from 60 to 90 °C with a plate reading every 0.2 °C. Standard curves were performed for each run with a genomic extract of the DiFi cell line at 1.8 ng·µL^−1^ of DNA. Each PCR run was carried out with no template control and positive control for each primer set. Positive controls were extracted from cell lines bearing *KRAS/BRAF* or *NRAS* point mutations, or synthetic DNA (Horizon dx). In each single run, negative and positive controls for each tested mutation were included and one standard curve was prepared. Validation of qPCR amplification was performed by melt curve differentiation. CfDNA mutation testing was done without any sensitivity cutoff. When point mutation was found in only one of the two replicates, it was confirmed in triplicate.

### 2.4 Design of cfDNA analyses

Changes in the total level of circulating cfDNA were analyzed in all serial plasma samples (*n* = 136). Quantification of cfDNA was obtained by amplifying a 67 bp length WT sequence of the *KRAS* gene. Pretreatment and after treatment plasma mutational analysis was performed (total number of plasma samples, *n* = 70). Point mutations analysis by IntPlex® ASB qPCR method included *KRAS* codons 12, 13, 61, 117, and 146, *NRAS* codons 12, 13, and 61, and *BRAF^V600E^*. Detection and quantification of *KRAS*, *NRAS,* or *BRAF* point mutations were done by targeting a short specific mutant sequence (< 100 bp length). In this analysis, mutant ctDNA referred to all ctDNA point mutation‐bearing sequences within one or more of the analyzed *RAS/BRAF* loci. Mutant ctDNA could be referred to as *RAS* mutant ctDNA where analysis focused solely on the quantification of *KRAS‐* and *NRAS*‐mutated ctDNA sequences. The mutation load corresponded to the proportion of mutant ctDNA fragments bearing a targeted mutation among all cfDNA fragments extracted from plasma. Likewise, the *RAS* mutation load was expressed as the proportion of *RAS* mutant ctDNA fragments bearing a targeted mutation in the *KRAS* and *NRAS* genes among all cfDNA fragments in a plasma extract.

### 2.5 Statistical analyses

Qualitative variables were described as percentages and quantitative variables as medians and interquartile ranges (IQR). Tumor response was defined according to RECIST 1.1 criteria. Progression‐free survival (PFS) was measured from the start of treatment to the date of progression or death from any cause. OS was defined as the time between the start of the treatment to death from any cause. Patients alive were censored at the last date they were known to be alive. The survival curves for OS were estimated using the Kaplan–Meier method, described with median and 95% CI, and compared using the log‐rank test. The associations between patient characteristics and outcomes were estimated using the Cox proportional hazard model, and hazard ratio (HR) was provided with their 95% CI. Proportional hazard assumptions were examined graphically by plotting the log minus log of survival. Restricted cubic spline methodology was used to estimate the association between continuous variables and OS in order to identify the best transformation to apply in Cox proportional hazard models and cutoff of interest. All analyses were performed using sas version 9.4 (SAS Institute, Cary, NC, USA) and r software version 2.15.2 (R Development Core Team, Vienna, Austria; http://www.r‐project.org). *P* values of < 0.05 were considered statistically significant, and all tests were two‐sided. Due to the exploratory setting of the study, *P* values were not corrected for multiple tests.

## 3 Results

Patient and treatment characteristics are summarized in Table 1. In populations of patients with and without cfDNA measurements, clinical characteristics, treatments, and outcomes were similar. Of the 43 patients, 23 were male. The median age was 62.1 years. A total of 39 patients (90.7%) had their primary tumor located on the left side of the colon or rectum. All patients were refractory (or intolerant) to available standard cytotoxic agents. The majority of patients (97.7%) were previously treated with an anti‐angiogenic agent (bevacizumab or aflibercept), while over 50% (22/43) received prior anti‐EGFR monoclonal antibody (cetuximab or panitumumab) therapy. The available tumor genotyping data showed mutation of *KRAS* for 16/43, mutation of *NRAS* for 3/29, *NRAS* unknown status for 14/43, mutation of *BRAF* for 5/35, and *BRAF* unknown status for 8/43. Tumor genotyping data were obtained either from resections (*n* = 27) or biopsies (*n* = 13) of primary tumors (*n* = 18) or metastases (*n* = 7). Corresponding cfDNA genotypes are shown in Table S1. Median treatment duration was 8.29 weeks (IQR, 4.14–15.14). Median OS for all patients was 5.3 months (3.7–8.6). The actual regorafenib median dose of the prescribed dose was 82.5%. The median cfDNA concentration at baseline (the cfDNA population) was 21.18 ng·mL^−1^ (IQR, 7.81–62.80).

**Table 1:** Patient and treatment characteristics. ECOG PS, Eastern Cooperative Oncology Group performance status.

	TEXCAN population *N* = 55	Population without cfDNA analysis *n* = 12	Population with cfDNA analysis *n* = 43
Median age, years (IQR)	62.7 (52.6–68.9)	67.7 (59.7–70)	62.1 (50.5–68.3)
Gender			
Male	30 (55.5%)	7 (58.3%)	23 (53.5%)
Female	25 (45.5%)	5 (41.7%)	20 (46.5%)
ECOG PS			
0	17 (30.9%)	4 (33.3%)	13 (30.2%)
1	38 (69.1%)	8 (66.7%)	30 (69.8%)
Primary site of disease			
Right colon	6 (10.9%)	2 (16.6%)	4 (9.3%)
Left colon	49 (89.1%)	10 (83.4%)	39 (90.7%)
Archival tumor genotype status			
KRAS WT	34	7	27
*KRAS* mutant	21	5	16
NRAS WT	34	8	26
*NRAS* mutant	3	0	3
Missing	18	4	15
BRAF WT	37	7	30
*BRAF* mutant	6	1	5
Missing	12	4	8
Time from diagnosis of metastases < 18 months	12 (21.8%)	3 (25%)	9 (20.9%)
Previous anti‐EGFR treatment	28 (50.9%)	6 (50%)	22 (54.1%)
Previous anti‐angiogenic treatment	54 (98.2%)	12 (100%)	42 (97.7%)
Regorafenib treatment			
Treatment duration, weeks (IQR)	8.29 (4.14–16.29)	12.14 (4–21.79)	8.29 (4.14–15.14)
Actual dose / planned dose	84.5%	100%	82.5%
Median OS, months (95% CI)	5.32 (3.7–8.6)	3.9 (1.7–17.1)	5.3 (3.7–8.6)

### 3.1 Relationship between circulating DNA markers and outcomes

**Fig. 2:**
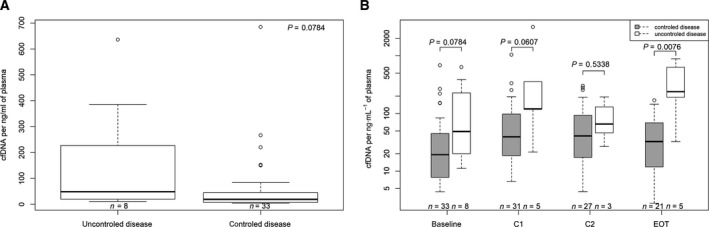
Circulating cell‐free DNA (cfDNA) and tumor response. (A) Baseline cfDNA and tumor control. (B) Dynamic changes in cfDNA concentrations throughout treatment duration. The boxplot shows medians with IQR (Q1–Q3), and whiskers represent the range within the 1.5 IQR. Test for comparison of median between groups is done by the Wilcoxon test. C1, cycle 1; C2, cycle 2; EOT, EOT.

**Fig. 3:**
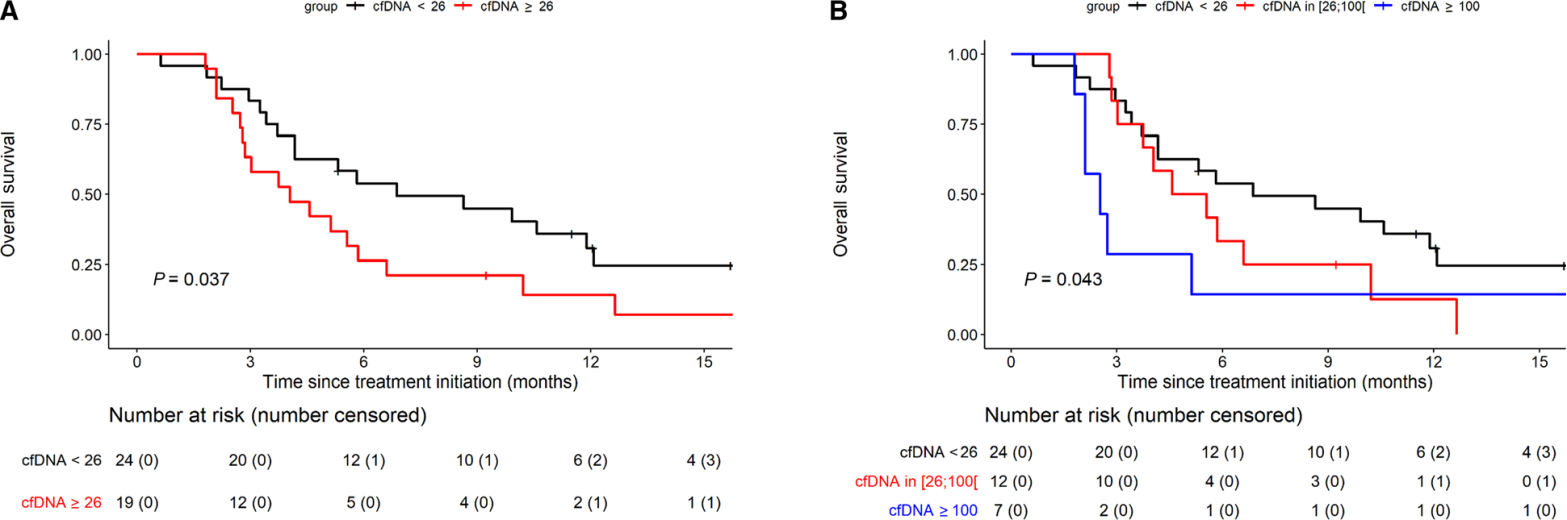
OS according to the baseline circulating cell‐free (cfDNA) concentrations. (A) Baseline cfDNA at 26 ng·mL^−1^ cutoff threshold. (B) Baseline cfDNA at 26, 26–110, and ≥ 100 ng·mL^−1^ cutoff thresholds. Survival curves are represented according to thresholds of the total cfDNA concentration (ng·mL^−1^ of plasma) determined at baseline. OS was estimated using the Kaplan–Meir method, described with median and 95% CI, and compared using the log‐rank test.

Among patients evaluable for tumor response (*n* = 41), those with tumor control as the best response (*n* = 33) had a median baseline cfDNA concentration of 19.4 ng·mL^−1^ compared with 48.77 ng·mL^−1^ for those with tumor progression (*n* = 8; *P* = 0.0784; Fig. 2A). To assess the association between survival outcomes and total baseline cfDNA, we tested three different cutoff values. The 26 ng·mL^−1^ threshold was chosen based on our previous observation that mCRC patients with cfDNA concentration > 26 ng·mL^−1^ had a lower OS than those with a cfDNA concentration below this threshold [29]. Figure 3A shows that patients with baseline cfDNA level of > 26 ng·mL^−1^ had shorter OS than patients with higher cfDNA cutoff level (the median OS 4.0 vs 6.9 months, respectively; log‐rank *P* = 0.0366). Similar findings were observed with the 65 ng·mL^−1^ threshold (i.e., third IQR of the cfDNA concentration distributions of the analysis) and 100 ng·mL^−1^ threshold (Table 2, Fig. 3B). Interestingly, we found that the probability of being alive at 3 months (a key inclusion criteria in mCRC clinical trials) was 83.3% (95% CI: 66.61–92.14) and 28.6% (95% CI: 4.11–61.15) in patients with the cfDNA level of < 100 and ≥ 100, respectively. Given any association between OS and cfDNA concentrations in its continuous form, we evidenced a positive correlation between these two, the higher the baseline cfDNA concentration, the higher the risk of death (Fig. S1).

**Table 2:** Univariate Cox regression analysis for survival according to baseline cfDNA.

	Baseline cfDNA (ng·mL^−1^)	*N* (events)	HR	95% CI	*P* value
OS	< 100	36 (29)	1		0.0355
	≥ 100	7 (7)	2.48	1.06–5.77	
	< 26	24 (18)	1		0.0407
	≥ 26	19 (18)	2.02	1.03–3.95	
	< 65	33 (26)	1		0.0458
	≥ 65	10 (10)	2.12	1.01–4.45	
	< 26	24 (18)	1		0.0534
	26–100	12 (11)	1.67	0.78–3.62	0.19
	≥ 100	7 (7)	2.93	1.20–7.17	0.0188
	Log	43(36)	1.26	1.01–1.56	0.0371
PFS	< 26	24 (22)	1		0.1947
	≥ 26	19 (19)	1.51	0.81–2.83	
	< 65	33 (31)	1		0.4507
	≥ 65	10 (10)	1.32	0.64–2.71	
	< 100	36 (4)	1		0.3683
	≥ 100	7 (7)	1.47	0.64–3.40	
	< 26	24 (22)	1		0.4115
	26–100	12 (12)	1.45	0.71–2.94	0.311
	≥ 100	7 (7)	1.66	0.69–4.00	0.2589
	log	43 (41)	1.2	0.97–1.49	0.0946

**Fig. 4:**
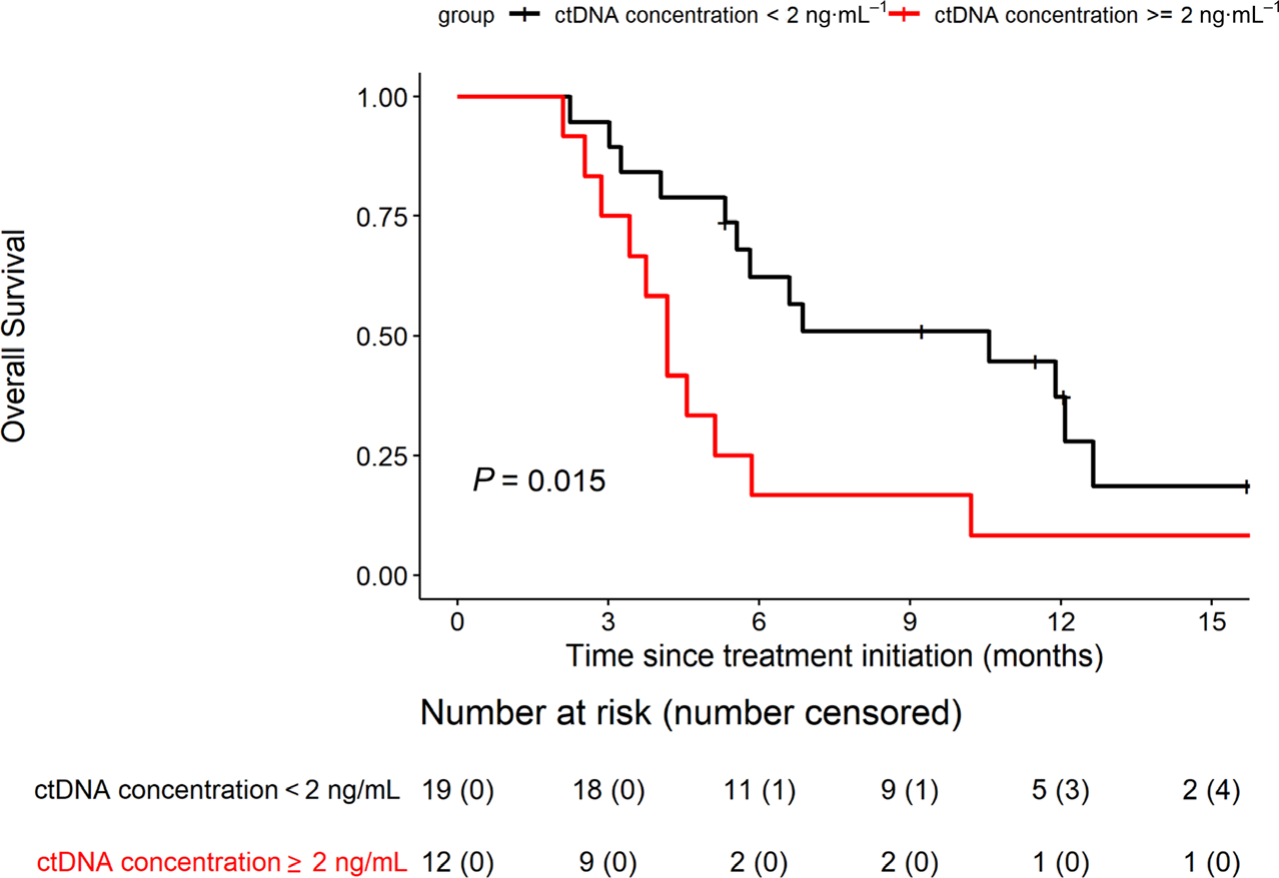
OS according to ctDNA concentrations (threshold 2 ng·mL^−1^) in patients with *RAS*‐mutated tumors OS was estimated using the Kaplan–Meir method, described with median and 95% CI, and compared using the log‐rank test.

When assessing the *RAS* mutant ctDNA concentrations, we observed that mCRC patients with baseline *RAS* mutant ctDNA concentration of > 2 ng·mL^−1^ (the threshold established from the relationship between *RAS* mutant ctDNA concentrations and the risk of death using the restricted cubic spline regression analysis; data not shown) had shorter OS than those with below the threshold (log‐rank *P* = 0.0154; Fig. 4). To a lesser extent, patients with a *RAS* mutation load of > 6% had shorter OS than the rest of patients (log‐rank *P* = 0.0200; Fig. S2). No relationship was detected between PFS and any of the circulating DNA markers studied (Table 2).

### 3.2 Changes in cfDNA concentration during treatment

We did not observe any meaningful variation of cfDNA along treatment duration, but an early increase in total cfDNA concentration from baseline to day 15 of cycle 1 (paired analysis, Wilcoxon signed‐rank test, *P* = 0.0004; Fig. S3). Moreover, patients without disease control had significantly higher cfDNA concentrations at the EOT than those who had stable disease as the best response (*P* = 0.0076; Fig. 2B). In fact, at baseline, on day 15 of cycle 1, on day 15 of cycle 2, and at EOT, patients with tumor control as best response had the following median cfDNA concentrations: 19.4, 39.29, 40.76, and 36.67 ng·mL^−1^, respectively. Also, patients who progressed had the following median cfDNA concentrations: 48.77, 119.73, 65.59, and 239.2, respectively (Fig. 2B).

### 3.3 Mutational analysis

At baseline, 33/43 patients (76.7%) showed *RAS/BRAF* mutations, of whom 30 had only *RAS* point mutations, two had *BRAF^V600E^* point mutations, and one had both *KRAS* and *BRAF* mutations (Table S1). At the EOT, mutation analysis of ctDNA of 27 patients showed *RAS* and/or *BRAF* mutations in 25 of theme (92.6%). Plasma analysis showed a high clonal heterogeneity of these patients' tumors with 10/33 and 12/25 mutated patients showing multiple concurrent *RAS/BRAF* mutations prior and after regorafenib treatment, respectively. Paired analysis revealed that for 23 patients whose ctDNA was mutated at baseline, ctDNA remained mutated at the EOT (including five patients with one or more newly point mutations detected during treatment that were not detectable at baseline). Two patients with WT tumors acquired mutations by the EOT (Table S1). Only for two patients, the *RAS/BRAF* status remained WT at the end of regorafenib treatment. Overall, we found that the mutational status changed from baseline to the EOT in 8/27 (29.6%). These changes did not translate into different outcomes from those identified at the baseline (data not shown). The *RAS/BRAF* mutation analysis performed on archival tumor tissue (gold standard) showed 13 false negatives and four false positives when compared to plasma analysis performed prior to the initiation of treatment (Table S1). The accuracy rate (*RAS/BRAF* mutant vs *RAS/BRAF* nonmutant patients) for the *RAS/BRAF* mutational status between the two assays was 60.5%, with a sensitivity of 83.3% and a specificity of 32%. These comparative values are similar to those previously reported [15], confirming the high sensitivity of the IntPlex detection test. The high number of false‐negative samples can be due to several reasons: (a) the intra‐ and intertumoral heterogeneity of tumors, (b) the clonal evolution of tumors over time, which may be accentuated by the different types of treatment given to patients, or (c) the lack of analytical sensitivity of the sequencing techniques used on the tumor tissue.

## 4 Discussion

Here, we present the ancillary analysis of the phase II TEXCAN study population of regorafenib‐treated mCRC patients. In this small, but prospective series, we found that the total cfDNA, *RAS* mutant ctDNA, and to a lesser extent the mutational load were correlated with OS, regardless of whether this relationship was studied as classes or as continuous variables. For example, patients with total baseline cfDNA ≥ 26 ng·mL^−1^ have double risk of death compared with those patients who have baseline cfDNA lower than 26 ng·mL^−1^. Although pre‐analytical requirements are becoming increasingly stringent with time [24], this threshold dichotomizes nicely the outcome of mCRC patients treated with regorafenib. This observation is similar to that of our earlier study [29]. Such inverse correlation between circulating DNA biomarkers and outcome in patients treated with regorafenib has also been reported by others using different methods and various data sets [7, 30, 31, 32, 33]. We could not, however, define whether this relationship is driven by the regorafenib use due to the design of our trial, which lacks a control group of patients not treated with regorafenib. Conversely to what has been previously reported [31, 34], we were unable to find a clear relationship between circulating DNA biomarkers and PFS. Similarly, although we did not identify any clear relationship between the total cfDNA level at baseline and disease control as the best response, a trend was observed. This could be explained by a weak impact of regorafenib on the tumor due to brevity of treatment duration related to adverse events and/or lack of the efficacy in this population of heavily pretreated patients for whom the best response is often tumor stabilization.

In line with other reports [30, 31], we found the cfDNA level to be increased 2 weeks after regorafenib onset. It has been suggested that this may be related to the release of cfDNA into the bloodstream secondary to the toxic effects of regorafenib on normal tissues [30]. Vandeputte *et al*. in their study of 20 mCRC patients treated with regorafenib, suggested that cfDNA and mutant ctDNA evolve independently with different patterns of dynamics [30]. Although it is always difficult to do cross‐studies comparison using different assays, conditions, and sets of patients, we hypothesize that this discrepancy could be related to the interstudy variability in the number of patients being sensitive to treatment (toxicity on normal tissues and/or an antitumor effect) to regorafenib.

As anticipated, we identified several mutational changes in circulating cfDNA (Table S1) within 8 weeks from the starting dose of regorafenib (i.e., the median duration of regorafenib treatment). Although these changes did not translate into different outcomes, their unprecedented rapid nature in our series is impressive. Based on this observation and of that from our previous report in a similar group of patients undergoing other experimental therapies [21], we suggest that these mutational changes are more likely related to the clinical characteristics of these heavily pretreated mCRC patients rather than to the regorafenib itself.

This study is exploratory and has some limitations, and the data should be interpreted carefully. Given the exploratory nature of this analysis, *P* values were not corrected for multiple testing. The sample size for this analysis was small could not counterbalance the lack of power due to the loss of 12 patients (22%) of the native TEXCAN population. Moreover, as regorafenib is more likely to be cytostatic than cytotoxic and as the treatment duration is very short, it would be unreasonable to expect meaningful tumor shrinkage with a sharp decrease in circulating DNA biomarkers in the setting of heavily pretreated mCRC patients.

## 5 Conclusion

In this ancillary study of the prospective TEXCAN phase II trial, we confirmed the potential benefit of cfDNA analysis prior to regorafenib treatment as a suitable method to identify mCRC patients more likely to have the best outcomes. Given our setting and inclusion of heavily pretreated mCRC patients, further investigation involving a larger validation cohort is needed to determine whether or not the 26 ng·mL^−1^ cutoff for cfDNA analysis is the most optimal to discriminate between patients with poor, good, and the best prognoses.

## Conflict of interest

TA has served in a consulting/advisory role and/or received honoraria from Amgen, Bristol‐Myers Squibb, Chugai, Clovis, Gritstone Oncology, Haliodx, MSD Oncology, Pierre Fabre, Roche/Ventana, Sanofi, Servier, and Tesaro/GSK, and has received travel, accommodation, and expenses from Roche/Genentech, MSD Oncology, and Bristol‐Myers Squibb. MJ has received travel, accommodation, and expenses from Roche/Genentech, and Pfizer. DV has reported a consulting role for GERCOR, HalioDX, Incyte, OSE Immunotherapeutics, Janssen‐Cilag, Pfizer, and CellProthera. CT has received travel expenses from Roche, Servier, Bayer, and BMS; has received research funding from Bayer; and has reported advisory board fees from Bayer, Roche, and Amgen. CL has reported a consulting/advisory role and receiving honoraria from MSD, Halozyme, Roche, Celgene, Amgen, and Servier, and has received travel accommodation from MSD and Roche. ART is a DiaDx stockholder. TM has disclosed research funding from Roche and Amgen; has received honoraria from Amgen, Sanofi, Bristol‐Myers Squibb, and Sandoz; has reported travel, accommodation, and expenses from Amgen and Merck. AA has reported a consulting/advisory role and/or receiving honoraria from Bristol‐Myers Squibb, MSD Oncology, and Servier; and has received research funding from Bayer Pharmaceuticals. All remaining authors have declared no conflicts of interest.

## Author contributions

ART and TA designed the study. BP and ART developed the methodology. BP, SA, AB, BR, and CS did the experiments under the supervision of ART. TA, IT, CT, MJ, TM, and CL contributed to the blood and clinical collections. JH and DV did the statistical analyses. AA and BP analyzed the data and prepared the manuscript. All of the authors (BP, TA, JH, IT, CT, MJ, TM, CL, SA, AB, BR, CS, DV, ART, and AA) discussed the results and approved the manuscript.

## Supporting information

**Fig. S1**. Risk of death according to baseline cfDNA concentrations. The dotted lines represents the 95% confidence interval for the risk of death according to cfDNA concentration at baseline (continuous line).
**Fig. S2**. Overall survival according to mutation load (threshold 6%).
**Fig. S3**. Changes of cfDNA concentrations during treatment. Serial concentration of circulating cfDNA (ng·mL^−1^ of plasma) was determined using the IntPlex ASB qPCR method targeting a 67 bp‐length wild‐type sequence of the *KRAS* gene. A significant increase of cfDNA concentrations from baseline to day 15 of cycle 1 was observed; test for comparison of cfDNA medians between baseline and cycle 1 with paired data (Wilcoxon signed rank test, *P* = 0.0004). EOT, End Of Treatment.
**Table S1**. Tumor genotyping analysis. Comparison of *KRAS*, *BRAF,* and *NRAS* genotypes in archival tissues and blood samples taken prior and at the end of regorafenib treatment.

## Data Availability

The data are not available in a public database or repository. The data sets generated and analyzed in this study are available from the corresponding author on reasonable request.
