# Supplementary material for: Monitoring levels of circulating cell‐free DNA in patients with metastatic colorectal cancer as a potential biomarker of responses to regorafenib treatment
Source: Mol Oncol. 2021 Jun 22;15(9):2401–11. doi: 10.1002/1878-0261.12972 (PMC8410523; doi:10.1002/1878-0261.12972)
Supplement: Supplementary file 1 — Fig. S1. Risk of death according to baseline cfDNA concentrations. The dotted lines represents the 95% confidence interval for the risk of death according to cfDNA concentration at baseline (continuous line). Fig. S2. Overall survival according to mutation load (threshold 6%). Fig. S3. Changes of cfDNA concentrations during treatment. Serial concentration of circulating cfDNA (ng·mL−1 of plasma) was determined using the IntPlex ASB qPCR method targeting a 67 bp‐length wild‐type sequence of the KRAS gene. A significant increase of cfDNA concentrations from baseline to day 15 of cycle 1 was observed; test for comparison of cfDNA medians between baseline and cycle 1 with paired data (Wilcoxon signed rank test, P = 0.0004). EOT, End Of Treatment. Table S1. Tumor genotyping analysis. Comparison of KRAS, BRAF, and NRAS genotypes in archival tissues and blood samples taken prior and at the end of regorafenib treatment. [file MOL2-15-2401-s001.docx]

**Supplementary information**

**Fig S1.** Risk of death according to baseline cfDNA concentrations

The dotted lines represent the 95% confidence interval for the risk of death according to cfDNA concentration at baseline (continuous line).

**Fig S2**. Overall survival according to mutation load (threshold 6%)

**Fig. S3**. Changes of cfDNA concentrations during treatment

Serial concentration of circulating cfDNA (ng/mL of plasma) was determined using the IntPlex ASB Q-PCR method targeting a 67 bp-length wild-type sequence of the *KRAS* gene. A significant increase of cfDNA concentrations from baseline to day 15 of cycle 1 was observed; test for comparison of cfDNA medians between baseline and cycle 1 with paired data (Wilcoxon signed rank test, *P* = 0.0004).

EOT, End Of Treatment

**Table S1.** Tumor genotyping analysis

Comparison of *KRAS*, *BRAF,* and *NRAS* genotypes in archival tissues and blood samples taken prior and at the end of regorafenib treatment.

**Fig. S1.** Risk of death according to cfDNA concentration at baseline


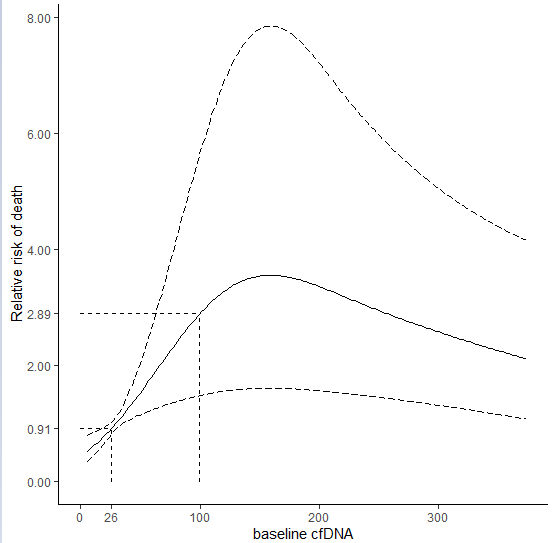


**Fig. S2.** Overall survival according to tumor mutation load (threshold 6%)


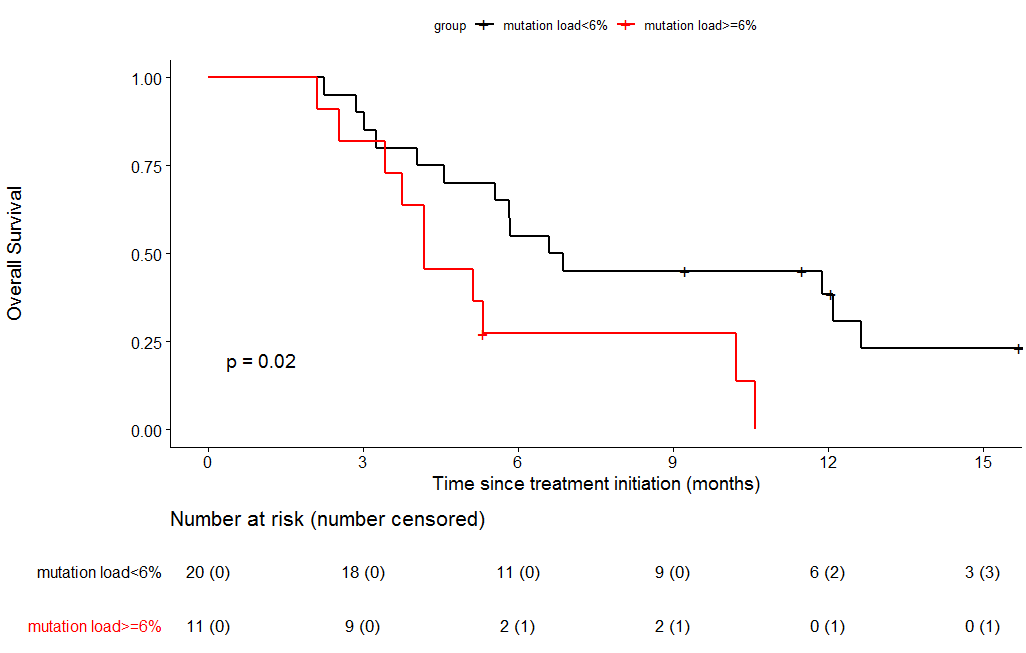


**Fig. S3.** Changes in cfDNA concentrations during treatment


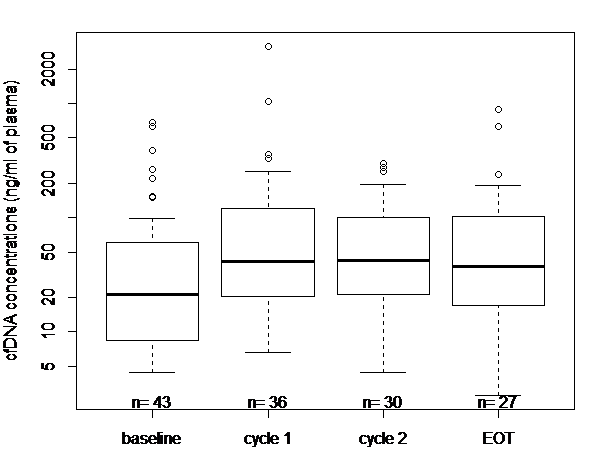


**p=0.0004**

**Table S1.** Tumor genotyping analysis
